# The Sickle Africa Data Coordinating Centre (SADaCC): a data science hub for interdisciplinary sickle cell disease research and training

**DOI:** 10.1093/database/baag007

**Published:** 2026-02-20

**Authors:** Ambroise Wonkam, Nchangwi Syntia Munung, Mario Jonas, Wilson Mupfurirwa, Arthemon Nguweneza, Kevin Esoh, Chandre Oosterwyk-Liu, Zimkita Magangana, Khuthala Mnika, Valentina Ngo Bitoungui, Martha Kamkuemah, Kambe Banda, Nabeelah Samie, Jade Hotchkis, Victoria Nembaware, Andre-Pascal Kengne, Nicola Mulder

**Affiliations:** Division of Human Genetics, Faculty of Health Sciences, University of Cape Town, Anzio Road, Observatory, 7925 Cape Town, South Africa; McKusick-Nathans Institute and Department of Genetic Medicine, Johns Hopkins University School of Medicine, 773 N. Broadway, Baltimore, MD 21205, United States; Division of Human Genetics, Faculty of Health Sciences, University of Cape Town, Anzio Road, Observatory, 7925 Cape Town, South Africa; Division of Human Genetics, Faculty of Health Sciences, University of Cape Town, Anzio Road, Observatory, 7925 Cape Town, South Africa; Division of Human Genetics, Faculty of Health Sciences, University of Cape Town, Anzio Road, Observatory, 7925 Cape Town, South Africa; Division of Human Genetics, Faculty of Health Sciences, University of Cape Town, Anzio Road, Observatory, 7925 Cape Town, South Africa; Division of Human Genetics, Faculty of Health Sciences, University of Cape Town, Anzio Road, Observatory, 7925 Cape Town, South Africa; McKusick-Nathans Institute and Department of Genetic Medicine, Johns Hopkins University School of Medicine, 773 N. Broadway, Baltimore, MD 21205, United States; Division of Human Genetics, Faculty of Health Sciences, University of Cape Town, Anzio Road, Observatory, 7925 Cape Town, South Africa; Division of Human Genetics, Faculty of Health Sciences, University of Cape Town, Anzio Road, Observatory, 7925 Cape Town, South Africa; Division of Human Genetics, Faculty of Health Sciences, University of Cape Town, Anzio Road, Observatory, 7925 Cape Town, South Africa; Division of Human Genetics, Faculty of Health Sciences, University of Cape Town, Anzio Road, Observatory, 7925 Cape Town, South Africa; Department of Microbiology, Hematology and Immunology, University of Dschang, PO Box 96, Dschang, Cameroon; Division of Human Genetics, Faculty of Health Sciences, University of Cape Town, Anzio Road, Observatory, 7925 Cape Town, South Africa; Division of Human Genetics, Faculty of Health Sciences, University of Cape Town, Anzio Road, Observatory, 7925 Cape Town, South Africa; Division of Human Genetics, Faculty of Health Sciences, University of Cape Town, Anzio Road, Observatory, 7925 Cape Town, South Africa; Division of Human Genetics, Faculty of Health Sciences, University of Cape Town, Anzio Road, Observatory, 7925 Cape Town, South Africa; Division of Human Genetics, Faculty of Health Sciences, University of Cape Town, Anzio Road, Observatory, 7925 Cape Town, South Africa; African Population & Health Research Centre (APHRC), Manga Close, Kirawa Road, Nairobi, Kenya; Division of Human Genetics, Faculty of Health Sciences, University of Cape Town, Anzio Road, Observatory, 7925 Cape Town, South Africa; Division of Computational Biology, Faculty of Health Sciences, University of Cape Town, Anzio Road, Observatory, 7925 Cape Town, South Africa

## Abstract

Sickle cell disease (SCD) is one of the most prevalent monogenic disorders worldwide, with the highest burden in Africa, where ~75% of the 7.74 million global cases occur. Scientific progress in understanding its epidemiology, clinical heterogeneity, and treatment outcomes has been constrained by heterogeneous, non-standardized, and non-interoperable datasets that limit data integration and cross-country analyses. To address this, the Sickle Africa Data Coordinating Centre (SADaCC) was established as the data science hub of the SickleInAfrica consortium to support the development and expansion of Pan-African SCD registry. SADaCC now coordinates one of the largest patient-consented SCD datasets globally, with data from over 40 000 persons living with SCD in seven countries (Ghana, Mali, Nigeria, Tanzania, Uganda, Zambia, and Zimbabwe) within the Sickle Pan-African Research Consortium (SPARCo), as well as genomic data from SADaCC satellite sites in Cameroon, South Africa, and Malawi. The registry is built on FAIR-compliant architecture, the Sickle Cell Disease Ontology, and powered by a suite of digital platforms such as REDCap, NextCloud, RStudio, GitHub, Docker, and Jupyter. In partnership with SPARCo, SADaCC is also piloting a biobank that will link biospecimens with data in the registry to advance multi-omics research. Beyond infrastructure, SADaCC leads training and/or research in big data analytics, genomics, bioethics, implementation science, qualitative research, and psychosocial studies. Ethical, legal, and social considerations are embedded across all operations with emphasis on equitable intra-African collaboration and patient involvement in research. Looking ahead, SADaCC will integrate real-time data streams, AI-driven analytics, and multi-omics data to drive big data and genetic medicine research for SCD in Africa.

## Introduction

Sickle cell disease (SCD) is a group of severe inherited haemoglobin disorders caused by homozygous (HbSS) or compound heterozygous (HbSC, HbS/β-thalassemia) mutations in the β-globin gene (HBB), most notably the Glu6Val substitution, which leads to the production of abnormal haemoglobin (HbS) [1, 2]. Under hypoxic conditions, HbS polymerizes into rigid fibres, causing red blood cells to assume a sickle or banana shape. These distorted cells obstruct microcirculation and undergo premature haemolysis, triggering chronic haemolytic anaemia, vaso-occlusive crises, and progressive multi-organ damage involving the brain, kidneys, lungs, and other organs mortality [1, 3]. Collectively, these complications contribute to substantial morbidity, premature mortality, and psychosocial challenges [4].

More than 75% of the estimated 7.74 million persons living with SCD worldwide reside in sub-Saharan Africa [5, 6]. Yet, progress in SCD research and care across the region remains constrained by limited funding, fragmented data systems, and a lack of harmonized infrastructure for coordinated research. Clinical and research datasets for SCD are often scattered across hospitals, clinics, and research institutions, stored in non-digital or incompatible formats and lack standardization [5]. In the absence of longitudinal, interoperable datasets, researchers face significant challenges in modelling disease trajectories, identifying risk predictors, and developing evidence-based interventions.

Advances in digital health and data science, including mobile health (mHealth) platforms, cloud computing, and artificial intelligence (AI)-driven analytics, offer opportunities for real-time data collection, integration, and predictive modelling to improve SCD care. However, the adoption of these technologies across African research and health systems is limited due to persistent infrastructural, technical, regulatory, and funding challenges [7, 8]. Establishing a coordinated, multicounty data science centre can be a more feasible, cost-effective, and sustainable approach to overcoming these challenges and advancing SCD research in Africa.

To respond to the need for harmonized longitudinal SCD datasets [9, 10], the Sickle Africa Data Coordinating Centre (SADaCC) was established under the SickleInAfrica consortium, a Pan-African network dedicated to advancing SCD research and care [11, 12]. The primary mandate of SADaCC was to support the establishment of a centralized, patient-consented, and interoperable SCD registry to support epidemiological surveillance, clinical trials, and translational research in Africa [10]. To achieve this, SADaCC spearheaded the development of the Sickle Cell Disease Ontology (SCDO), a machine-readable, semantically rich framework that standardizes SCD-related clinical and research terminology [13, 14] and used it to design and harmonize the SickleInAfrica registry [15].

Today, SADaCC coordinates one of the world’s largest harmonized and interoperable SCD registries, encompassing data from over 40 000 persons living with SCD across seven African countries within the Sickle Pan-African Research Consortium (SPARCo) [16–19], along with satellite genomics sites in Cameroon, South Africa, and Malawi [20, 21]. Its success required substantial investment in data infrastructure, bioethics, implementation science, genomics, bioinformatics, multi-country project coordination, and multidisciplinary training. This paper describes SADaCC’s infrastructure, operational framework, and key achievements as a data science hub for SCD research and outlines its strategic vision to lead data-driven, interdisciplinary SCD research and training in Africa.

## Methodology: collaborative foundations and core functions of SADaCC

SADaCC was established as the data science and coordination hub of the SickleInAfrica consortium, building upon foundational collaborations initiated by the SCDO Working Group, originally a partnership between the H3Africa Bioinformatics Network (H3ABioNet) and the Sickle Pan-African Network [14, 22, 23]. Additional contributions from some projects within the Human Heredity and Health in Africa (H3Africa) consortium [24, 25] and the PhenX Toolkit initiative for standardized measures further strengthened the technical and methodological foundation for the establishment of SADaCC as a Pan-African SCD data science ecosystem. These early partnerships provided the much-needed expertise in ontology development, bioinformatics, bioethics, and data infrastructure to initiate the development of an SCD data science centre.

The SCDO played an instrumental role in establishing standardized, machine-readable terminologies for the SickleInAfrica registry, thereby enabling both semantic and syntactic interoperability across participating SPARCo sites [14]. Technical collaboration with H3ABioNet facilitated efforts towards data curation, integration, and computational analytics, while engagement with the H3Africa network [26] facilitated access and collaborations with an African-wide community of practice in genomics, bioethics, SCD, and data science. SADaCC works in close coordination with the SPARCo Clinical Coordinating Centre, the Data Science for Health and Innovation in Africa (DS-I Africa) Initiative, the African Society of Human Genetics, and the Department of Genetic Medicine at Johns Hopkins University, SADaCC also maintains close partnerships with the SPARCo Clinical Coordinating Centre, the DS-I Africa Initiative, the African Society of Human Genetics, and the Department of Genetic Medicine at Johns Hopkins University, leveraging their collective expertise in data governance, genomics, public engagement, bioinformatics, and statistical and qualitative data analysis to advance the work of the SickleInAfrica consortium [21, 27].

**Figure 1:**
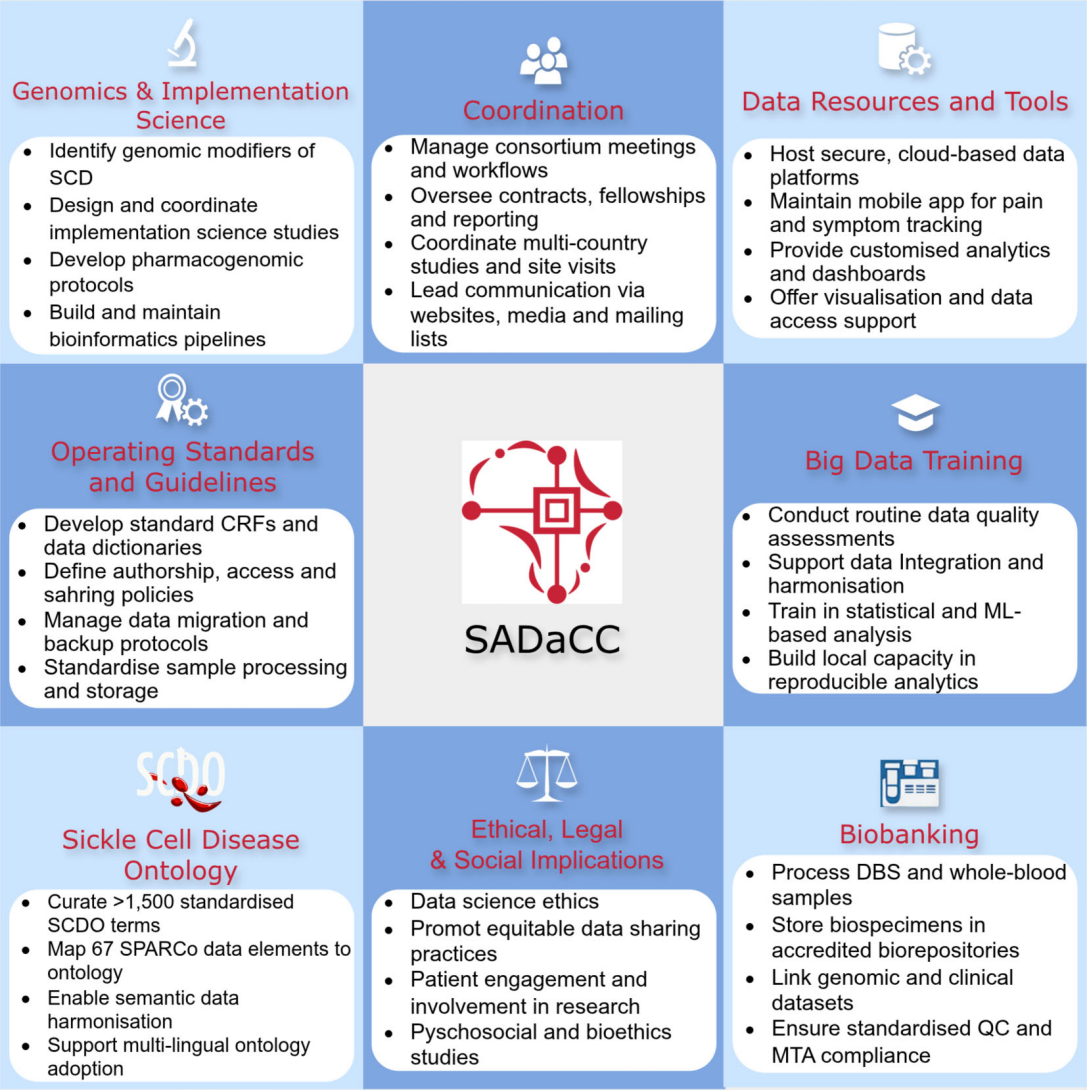
SADaCC-based activities in developing and expanding the SickleInAfrica registry.

The operations of SADaCC are guided by the following strategic objectives (Fig. 1):

Manage the centralized SickleInAfrica registry through secure, scalable platforms for data capture, transfer, and curation.
Provide advanced data analytics training, support, and digital tools to promote harmonized data management, visualization, and reproducible research.
Coordinate multi-country research and administrative activities, including developing standard operating procedures (SOPs), maintaining communication and reporting systems, and overseeing project monitoring and evaluation.
Support a broad research portfolio encompassing implementation science, clinical studies, genomics, bioethics, and psychosocial studies within SickleInAfrica.
Embed ethical, legal, and social implications frameworks across all data collection, sharing, and research activities.

## Results: achievements and growth of SADaCC

**Figure 2:**
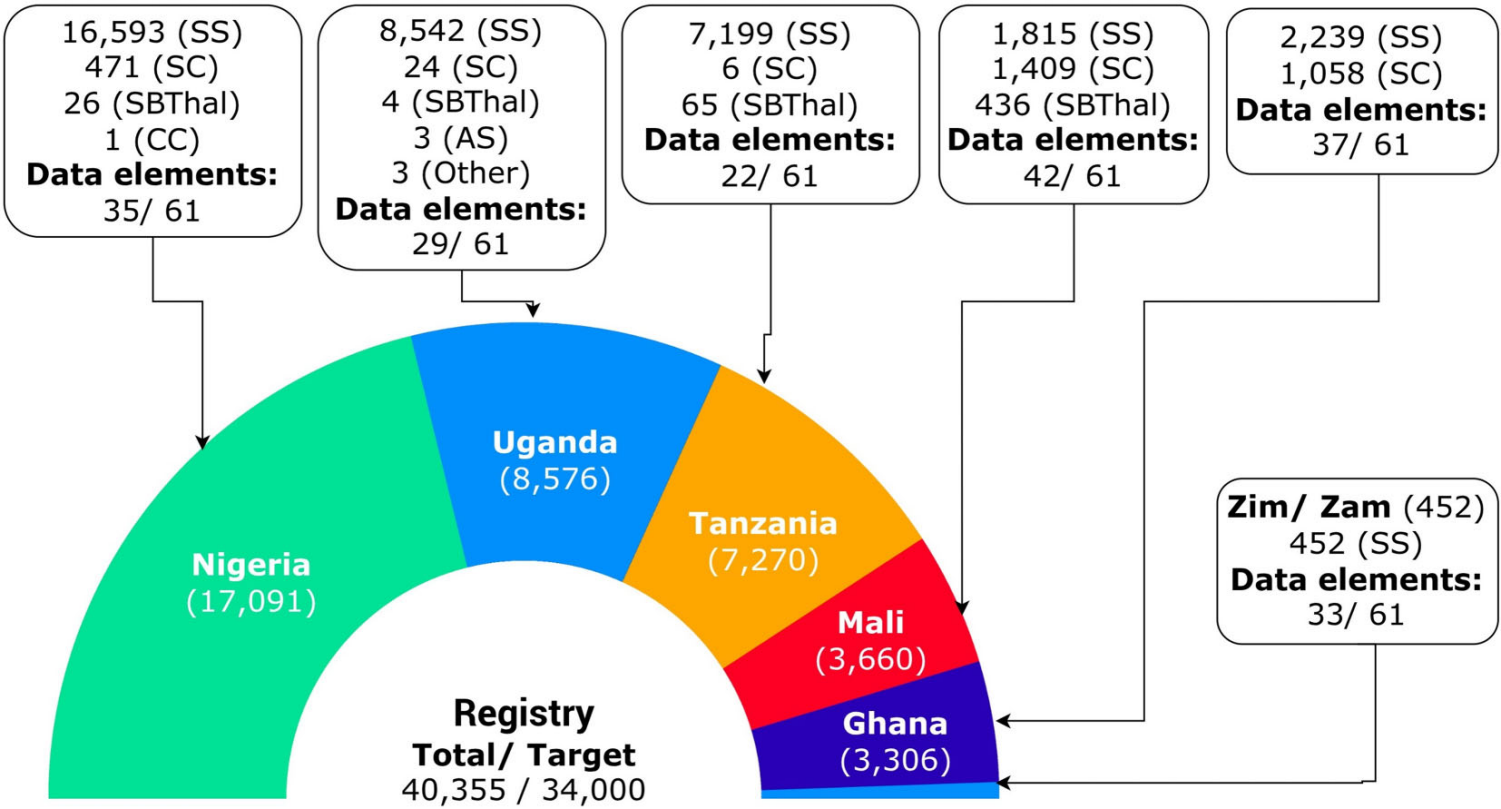
Available data in the SickleInAfrica registry from SPARCo clinical sites (November 2025). Zim/Zam: Zimbabwe and Zambia.

Since its inception, SADaCC has made substantial progress in establishing and expanding the SickleInAfrica registry, now recognized as arguably one of the largest longitudinal, patient-consented datasets on SCD globally. As of October 2025, the registry houses clinical and/or epidemiological data from more than 40 000 patients across Ghana, Mali, Nigeria, Tanzania, Uganda, Zambia, and Zimbabwe (Fig. 2), along with genomic data and biospecimens from Cameroon, Malawi, and South Africa. The registry captures both retrospective and prospective data, harmonized across demographic, clinical, laboratory, behavioural, and therapeutic domains, providing a unique resource for longitudinal and cross-country analyses. This comprehensive dataset now underpins an expanding portfolio of research, including multinational epidemiological studies, newborn screening pilots, pharmacogenomic analyses, and implementation research on malaria chemoprophylaxis and hydroxyurea use [15, 28, 29]. SADaCC is also piloting the establishment of an African SCD biorepository to support clinical and genomic studies into disease-modifying loci and molecular mechanisms influencing SCD outcomes [20, 21].

### Registry development and data infrastructure

At the heart of SADaCC’s mission is the design, implementation, and stewardship of the SickleInAfrica registry as an interoperable, longitudinal database built to integrate multi-country clinical and genomic data [15, 28]. To ensure optimal data management, integrity, and security, each SPARCo site is equipped with dedicated local servers for secure data storage, cleaning, and encrypted transfer to the central database hosted by SADaCC at the University of Cape Town, South Africa. A robust consortium-wide data governance framework standardizes all processes for data capture, validation, sharing, and archiving, in alignment with the FAIR (Findable, Accessible, Interoperable, and Reusable) principles. SADaCC further supports SPARCo sites through the development of SOPs, electronic case report forms (CRFs), and automated data migration and quality assurance pipelines, enabling consistent and reproducible data curation across countries. This ensures that registry operations meet global standards for data quality, traceability, and long-term sustainability.

### Data standardization and harmonization

To enable interoperability and large-scale analytics, SADaCC operationalizes the SCDO as a machine-readable semantic framework that supports automated data integration, computational querying, and harmonization across all SPARCo sites. During registry development, more than 1 500 site-specific variables were consolidated into a harmonized dataset comprising 61 core SCDO-aligned elements, spanning clinical, demographic, and laboratory domains. To streamline this process, natural language processing tools are employed to automatically map local variables from SPARCo sites to SCDO terms, followed by manual expert review to ensure precision and semantic consistency. Across the seven SPARCo sites, data collection varies between 22 and 42 of these 61 core variables, depending on clinical context and available infrastructure (Fig. 2). This ontology-driven model establishes a foundation for future integration of biospecimen metadata, genomic datasets, and digital health data, ultimately enabling AI- and machine learning-assisted predictive analytics, disease modelling, and decision support within SickleInAfrica projects.

### Data flow and curation

To ensure data integrity, harmonization, and analytical readiness, SADaCC has established a standardized, end-to-end data flow and curation framework governing the full lifecycle of registry data, from initial capture to integration, analysis, and reuse. Clinical and laboratory data are collected using REDCap-based CRFs aligned with the SCDO to ensure terminological consistency across sites. Each participating site performs local validation and de-identification before encrypted transmission to SADaCC. Within SADaCC, automated and manual quality control pipelines are used for routine data audits to identify duplicates, missing values, and outliers, alongside cross-validation against source documents to maintain accuracy. This multi-tiered approach ensures that data are standardized, high-quality and analysis-ready for research use across the consortium.

### Digital tech toolbox for streamlining data management and analysis

**Figure 3:**
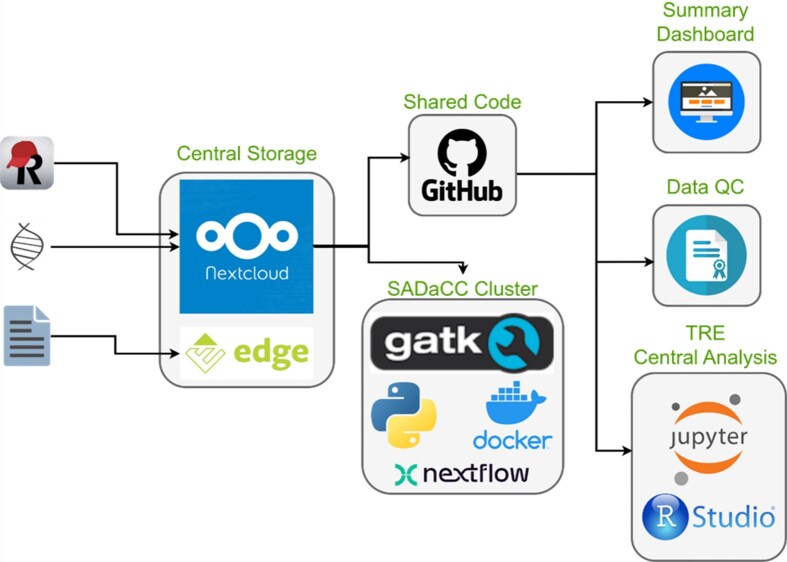
SADaCC digital infrastructure and processes for streamlining data management and analysis.

SADaCC ADaCC maintains a suite of interoperable, open-source, and cloud-based digital platforms designed to streamline data access, storage, visualization and computational analysis across SickleInAfrica (Fig. 3). These include

- NextCloud: a secure, cloud-hosted storage environment [30] for multisite data exchange and collaboration. This service is supported by the University of Cape Town.
- REDCap: a mobile and web-based application for real-time clinical data capture, even in low-connectivity settings [31].
- RStudio (via Ilifu Cloud): an integrated platform for statistical computing, predictive modelling, and collaborative analysis using high-performance computing infrastructure.
- Jupyter Notebooks: an interactive workspace for data exploration, visualization, and computational reproducibility [32].
- GitHub: a version-controlled repository that supports collaborative coding, reproducible workflows, and open data science practices.
- Docker: a containerization tool for reproducible deployment of analytic environments across heterogeneous systems [32].

Together, these tools overcome infrastructural challenges such as limited local storage and intermittent connectivity by allowing offline data capture with automated synchronization from tablets at SPARCo clinical sites. This digital ecosystem enhances transparency, reproducibility, and interoperability and ensures that registry data are efficiently managed and readily available for analysis across the consortium.

### Data analysis support and training to advance SCD research in Africa

SADaCC provides analytical, computational, and training support to strengthen data science capacity for SCD research across Africa. Researchers at SPARCo sites have remote access to a centralized RStudio environment hosted on the Ilifu High-Performance Computing Cluster at the University of Cape Town, which facilitates predictive modelling, phenotypic clustering, survival analysis, and longitudinal trend assessment. Increasingly, AI- and ML- driven algorithms will be applied to identify risk profiles, predict clinical complications, and support clinical decision-making. To promote transparency and reproducibility, standardized R and Python scripts developed by SADaCC and hosted on GitHub are shared across sites for harmonized analytical workflows.

To address skill gaps, strengthen research independence at SPARCo sites, and promote equitable intra-African collaborations, SADaCC has implemented structured in-person and virtual training programmes covering data capture (REDCap), programming (R, Python, SQL), big data analytics, ontology design, bioethics, implementation science, qualitative research, systematic and scoping reviews, and project management. Over the past 5 years, more than 50 trainees from seven African countries have completed SADaCC-supported fellowships and workshops, leading to improved data quality, reduced entry errors, and increased independent research outputs in newborn screening, pharmacogenomics, SCD epidemiology, and implementation science. The big data analytics and SCDO training workshops have eventually become flagship capacity-building programmes in Africa and offered more widely in partnership with the African Society of Human Genetics [33].

### Support for SCD research

SADaCC actively integrates data science, AI, and ML methodologies into ongoing SickleInAfrica research projects to enhance discovery and translational impact. Notable applications include Bayesian geostatistical modelling to estimate paediatric SCD prevalence in Africa [34]; regression analyses to examine child mortality and treatment response in Nigeria [35]; and nonlinear mixed-effects modelling to optimize hydroxyurea dosing strategies in diverse African populations [36]. Furthermore, genome-wide association study (GWAS) pipelines developed by SADaCC have identified HbF-modifying loci such as BCL11A and HBS1L-MYB, which are key to understanding disease modulation and informing future gene therapy strategies [21] SADaCC’s research portfolio also encompasses a broad spectrum of multidisciplinary studies, including support for the design of an implementation science projects evaluating point-of-care diagnostics using dried blood spots; observational studies on malaria chemoprophylaxis and hydroxyurea use; pharmacogenomic analyses; psychosocial and bioethics research, systematic and scoping reviews, and data governance [15, 20, 21, 29, 37–41]. In addition, SADaCC has established bioinformatics pipelines for GWAS and next-generation sequencing in African SCD populations [17, 18]. This ecosystem of research support spans study design, data curation, advanced analytics, dissemination, and embedding ethical, legal, and social considerations into all aspects of its scientific and data governance activities.

### Ethical legal and social issues

Ethical, legal, and social considerations are embedded throughout all SickleInAfrica activities [42, 43] with strong emphasis on equitable intra-African collaboration, patient involvement in research, and compliance with national and regional data protection laws [38, 42, 44]. As part of this objective, SADaCC, in collaboration with SPARCo sites and patient advocacy groups, co-developed consortium-wide data sharing agreements, authorship and publication policies, and informed consent templates [42]. To further guide inclusive data practices, we have proposed a road map for cultivating an equitable data sharing culture in African science initiatives centred on principles of data solidarity, federated analytics, and the possibility of social contracts for research data use in Africa [45, 46]. A recent shift towards participatory research governance has positioned SCD patient representatives as co-creators in defining research priorities and shaping data policy through the Patients as Partners in Sickle Cell Disease Research in Africa working group [44, 47].

In terms of data governance, SADaCC operationalizes a multi-layered data governance framework that safeguards participant privacy, data integrity, and equitable access while promoting responsible and inclusive data sharing. Although each SPARCo site retains responsibility for informed consent and participant verification, SADaCC conducts centralized data audit, combining automated and manual validation processes, and issues certificates of compliance to ensure adherence to data protection standards. In keeping with the principles of open and equitable data science, curated datasets are securely shared with contributing sites via NextCloud, enabling their use for research, quality improvement, and clinical audit activities. To further promote data transparency and utilization, interactive dashboards and visualization tools allow clinicians and researchers to transform registry data into actionable insights for patient care, health policy, and translational research.

### Biorepository development

In parallel with the SickleInAfrica registry, SADaCC is developing the SickleInAfrica Biorepository, a pilot initiative designed to integrate clinical, genomic, and phenotypic data to support multi-omics and translational research. The biorepository builds upon the experience and infrastructure of the H3Africa regional biorepositories in South Africa, Nigeria, and Uganda [48], enabling the centre to address logistical, ethical, and regulatory challenges associated with cross-border biospecimen sharing and governance. The pilot will see ~1000 biospecimens, including whole blood, plasma, serum, and extracted DNA, securely stored at designated biobanks under standardized protocols for sample processing and quality control, and associated to data in the SickleInAfrica registry.

### Project coordination

SADaCC functions as the project coordination nucleus for SickleInAfrica and performs this role in close collaboration with the SPARCo Clinical Coordinating Centre in Tanzania. SADaCC provides administrative, logistical, and scientific oversight for all consortium activities, including multi-country study coordination, grant management, fellowships, and early-career researcher exchanges. The centre also manages the SickleInAfrica website, digital communication channels, and social media platforms, with the goal of promoting visibility, knowledge dissemination, and stakeholder engagement. Additionally, SADaCC is responsible for workflow optimization, monitoring and evaluation of deliverables, and coordination of training and implementation programmes within SickleInAfrica. This has ensured that the consortium operations remain aligned, transparent, and outcome driven.

## Discussion: towards a data science hub for SCD research in Africa and globally

Over the past 8 years, SADaCC, supported by the US National Heart, Lung, and Blood Institute, the University of Cape Town, and SPARCo, has made substantial strides towards establishing a functional data science infrastructure for SCD research and innovation in Africa. Building on models such as the US Sickle Cell Disease Implementation Consortium [49, 50], SADaCC has systematically addressed long-standing challenges of fragmented data, inconsistent standards, and poor interoperability, factors that have historically constrained collaborative SCD research on the continent. Through an integrated framework for data harmonization, governance, and digital infrastructure, SADaCC has laid the foundation for an AI-ready ecosystem capable of driving big data research and precision medicine for SCD in Africa. A cornerstone of this progress is the SickleInAfrica registry (Fig. 2), which now includes over 40 000 patient-consented records across seven African countries, representing one of the most comprehensive longitudinal SCD datasets globally. Its digital toolbox, comprising REDCap, RStudio, NextCloud, Jupyter, GitHub, and Docker, provides an end-to-end infrastructure for secure data collection, curation, analysis, and reproducible research, enabling seamless collaboration across geographically dispersed teams. Future integration of mHealth tools, such as *SCD Warrior* (an in-house mobile application), will enable real-time capture of patient-reported outcomes and physiological metrics, further advancing adaptive and data-driven care models. These innovations mirror global trends in digital health and precision medicine for SCD [51, 52], and illustrate how African research institutions can serve as a testbed for scalable, data-driven management of chronic genetic diseases.

SADaCC’s contributions extend beyond infrastructure to human capital development. Through structured training programmes in data management, analytics, epidemiology, bioethics, and implementation science, the centre has empowered a new generation of African researchers, data scientists and clinicians to independently analyse, interpret, and translate large-scale datasets into actionable insights. Also, by embedding patient engagement within research design, consent, and oversight, SADaCC has institutionalized a patient-centred model of SCD research and empowered representatives of SCD patient support groups to have a voice as active participants and contributors in SickleInAfrica research [29, 42, 47].

Looking ahead, SADaCC envisions its evolution into an African Centre of Excellence for precision medicine and data-driven SCD research. This vision includes the integration of multi-omics datasets (genomic, proteomic, metabolomic) with mobile and wearable health data to enable AI- and ML-driven predictive analytics for early detection of complications and personalized care. The *SCD Warrior* app will in the future be used as a platform for continuous monitoring, real-time patient feedback, and data collection. To ensure sustainability, SADaCC is implementing a multi-pronged strategy that will involve partnering with other data science centres, SCD research programmes, universities, industry partners, philanthropic foundations, and global funders to support its long-term operations and expansion. These partnerships will also allow SADaCC to extend its data science framework to other chronic and genetic conditions.

## Conclusion: a data-driven future for SCD research in Africa

SADaCC is poised to become a leading data science centre of excellence for SCD data science research in Africa. The planned integration of real-time data streams, AI-powered analytics, and biorepository-linked genomic data into the centralized registry will enable advanced clinical trials, gene therapy studies, and predictive modelling.

Looking forward, SADaCC aims to

- Expand the SickleInAfrica registry to additional African countries and diaspora communities to enhance data diversity and representativeness.
- Integrate biorepository and multi-omics datasets with clinical and epidemiological data to enable biomarker discovery and mechanistic insights.
- Strengthen partnerships with AI developers, public health institutions, and global SCD networks to apply advanced computational methods to urgent clinical and policy challenges.
- Advance participatory data governance and patient-engaged research that prioritize equity and transparency.

As SADACC transitions into a mature SCD data science hub, it will play a pivotal role in applying AI and ML to develop predictive models for research and clinical decision-making such as forecasting pain crises, hospitalization risk, hydroxyurea responsiveness, and organ function. In doing so, SADaCC aims to showcase how collaborative African-led data science can advance research and development.

## Data Availability

Data sharing is not applicable to this article, as no datasets were generated or analysed during the current study.
